# Myofibril-Inducing RNA (MIR) is essential for tropomyosin expression and myofibrillogenesis in axolotl hearts

**DOI:** 10.1186/1423-0127-16-81

**Published:** 2009-09-03

**Authors:** Chi Zhang, Pingping Jia, Xupei Huang, Gian Franco Sferrazza, Gagani Athauda, Mohan P Achary, Jikui Wang, Sharon L Lemanski, Dipak K Dube, Larry F Lemanski

**Affiliations:** 1 Department of Biomedical Science, Florida Atlantic University, Boca Raton, FL 33431, USA; 2 Department of Radiation Oncology, Temple University School of Medicine, Philadelphia, PA 19140, USA; 3 Department of Anatomy and Cell Biology and The Cardiovascular Research Center, Temple University, Philadelphia, PA 19140, USA; 4 Department of Medicine, Upstate Medical University, Syracuse, NY 13210, USA; 5 Department of Biological and Environmental Sciences, Texas A&M University-Commerce, Commerce, TX 75429-3011, USA

## Abstract

The Mexican axolotl, *Ambystoma mexicanum*, carries the naturally-occurring recessive mutant gene 'c' that results in a failure of homozygous (c/c) embryos to form hearts that beat because of an absence of organized myofibrils. Our previous studies have shown that a noncoding RNA, Myofibril-Inducing RNA (MIR), is capable of promoting myofibrillogenesis and heart beating in the mutant (c/c) axolotls. The present study demonstrates that the *MIR *gene is essential for tropomyosin (TM) expression in axolotl hearts during development. Gene expression studies show that mRNA expression of various tropomyosin isoforms in untreated mutant hearts and in normal hearts knocked down with double-stranded MIR (dsMIR) are similar to untreated normal. However, at the protein level, selected tropomyosin isoforms are significantly reduced in mutant and dsMIR treated normal hearts. These results suggest that MIR is involved in controlling the translation or post-translation of various TM isoforms and subsequently of regulating cardiac contractility.

## Introduction

The Mexican axolotl, *Ambystoma mexicanum*, has proven to be a unique animal model in the study of cardiac development. The axolotl (a neotenous salamander) carries a naturally occurring recessive mutation, gene c, first discovered and characterized by Humphrey [1], which results in abnormal cardiac development in homozygous recessive "c/c" embryos. The mutant embryonic hearts develop, but fail to beat, making them distinguishable from normal embryonic hearts which start to beat at stage 35. The myocytes of the mutant hearts fail to form organized myofibrils and the embryos survive only to stage 42, the hatching stage, due to a lack of circulation.

Among the various myofibril structural proteins, tropomyosin has been shown by SDS-PAGE [2], radio-immunoassay [3], 2D gel electrophoresis [4] and confocal microscopy of whole hearts to be drastically reduced in the mutants [5-7]. Interestingly, other myofibril structural proteins such as actin, myosin and myosin binding protein C, however, were found to be at or near normal levels in the mutant hearts [8-10].

Using this animal model, Myofibril-Inducing RNA (MIR), a small bioactive RNA, was shown in previous studies to be able to restore tropomyosin protein synthesis, promote myofibrillogenesis, and initiate heartbeat in the mutant embryonic hearts in organ culture [11]. The MIR appears to function through its unique secondary structure since it is a non-coding RNA [7,11].

In mammals, birds and amphibians, altogether four different types of tropomyosin genes have been identified: alpha gene (TPM1), beta gene (TPM2), gamma gene (TPM3) and TM4 type gene (TPM4) [12]. More recently, TM4, a cytoskeletal tropomyosin, also has been associated with growth and regeneration in response to injury, disease state and stress in skeletal muscle of mouse and humans [13]. Moreover, in zebrafish embryos, a heart specific isoform of TM4 is essential for normal myofibril formation and developing a heartbeat [14]. In addition, it has been found recently that tropomyosin is likely necessary for actin filament formation in motile cells that employ lamellipodia and filopodia for locomotion [15]. Thus, tropomyosin appears to play a major role in actin filament modulation and contractility in both muscle and nonmuscle contractile systems. Striated muscle-specific alpha-tropomyosin is the predominant isoform in cardiac muscle, with low levels of beta-tropomyosin expressed during fetal development in the mouse heart [16]. In amphibian models, such as *Xenopus*, the cardiac muscle tropomyosins are synthesized from the alpha-TM and TM-4 genes [17]. In all of these animal models, the alpha-tropomyosin gene is the major contributor to the tropomyosin proteins in the cardiac myofibril structures.

There are at least three striated muscle isoforms of tropomyosin present in the axolotl. Two isoforms of tropomyosin cDNA have been identified which apparently are derived from the single alpha-tropomyosin gene (TPM1) through alternative splicing [18]. Spinner et al. [19] cloned another tropomyosin cDNA which is the product of a TM4 type tropomyosin gene from axolotl heart. Our results from the axolotl model of heart development are consistent with the findings in *Xenopus *in that both alpha (ATmC-1 and ATmC-2) and TM4 type (ATmC-3) tropomyosin transcripts are expressed in axolotl hearts [18,19].

In the present studies, we conducted a series of experiments to further understand the role of MIR in the expression of tropomyosin and myofibrillogenesis using our mutant axolotl heart model. First, we have cloned the full-length cDNA sequence of MIR. Sequence analyses suggest that MIR is a noncoding RNA molecule. However, functional studies indicate that the MIR is essential for tropomyosin expression as well as myofibrillogenesis in axolotl hearts during development at the level of translation or post-translation.

## Materials and methods

### Procurement of animal tissues

The embryos used in the study were derived from adult animals maintained in our colony. We followed NIH Guidelines for the Care and Use of Laboratory Animals and all animal protocols were approved by the Institutional Animal Care and Use Committee. Embryos at stages 35-38 were collected and dissected in Steinberg's solution (SS) as described previously [20]. The inner thoracic cavities were exposed and the hearts removed and used in the various bioassays.

### Bioassays with cationic liposome transfection and confocal microscopy

Liposome reagents (0.1-0.16 μg/μl) and MIR sense (500 nt) (0.022 μg/μl), antisense RNA (500 nt) (0.022 μg/μl) or dsRNA (500 bp, 0.002 μg/μl) were diluted in SS without antibiotics for 30-45 minutes at room temperature (RT). The two solutions were mixed and drops of transfection solution were prepared. The hearts are transferred with SS into drops containing the transfection solution for a total volume of 20 μl and appropriate concentrations of RNAs (7 ng/μl) or double-stranded RNA (4 ng/μl), and lipofectin. 10× of SS was added to the transfection medium to dilute the liposome reagents after 24 hours and the hearts were cultured for an additional 2 days (for mutant hearts + MIR RNA) or an additional 9 days (for normal hearts + dsRNA) in a 17°C incubator. The hearts were stained for immunofluorescent observation and analyzed by confocal microscopy following our previously published procedures [7,21,22].

### Synthesis of double-stranded MIR

Sense and antisense MIR are synthesized in vitro using T7 RNA polymerase (Ambion, TX) on PCR generated MIR cDNA templates with T7 promoter added to either 5' or 3' end sequences. 20 μg of both sense and antisense RNA are mixed in annealing buffer (pH 7.4) and denatured at 68°C for 5 minutes. Sense and antisense RNAs are annealed to each other to form dsRNA by gradually reducing the temperature to 25°C. The products were run on 1.5% agarose gels and the dsRNA was recovered from the gels. FITC-labeled dsRNA was synthesized as described above but using 1:3 FITC-conjugated UTP and unlabeled UTP for single-stranded RNA transcription.

### Real-time RT-PCR

Stage 36/37 normal and mutant embryonic hearts are explanted into 15 μl droplet cultures of Steinberg's buffered salt solution containing antibiotics [20]. Mutant hearts are treated with 40 ng/heart MIR sense (166 nt) or MIR antisense (166 nt) and incubated at 14°C for 36 hours or 72 hours. Each treatment group consisted of 40 hearts. As controls, 40 normal and 40 mutant hearts are left untreated. The total RNA is extracted from the axolotl hearts using TRI Reagent (Sigma). Reverse transcription is performed using the Thermoscript RT system from Invitrogen, CA. Quantitative PCR is performed in a Lightcycler system using a Roche's Fast Start SYBR Green I Kit, following our published methods [23]. The primers were designed by the Primer 3 program from the Massachusetts Institute of Technology as listed in the Table 1.

**Table 1:** Primers used for different genes in real time RT-PCR experiments.

Gene	Forward/Reverse	Primer Sequence	Product Size
ATmC-3	Forward	5'-ggagcttgaccatgcgctgaa	200

ATmC-3	Reverse	5'-tgagaaccgacacaaagcaagagg	

ATmC-exon2a	Forward	5'-gcgtgacagggtgctggatgaac (P5)	154

ATmC-exon2b	Forward	5'-tgaagggtaccgaggacgagttgga (P3)	

ATmC-exon3	Reverse	5'-ccagacgctcctgagcacgatcc (P4)	

ATmC-exon345	Forward	5'-gagcgtctggccacagccctaca (P1)	233

ATmC-exon345	Reverse	5'-tcttccgcacgctccagatcacc (P2)	

ATm5-	Forward	5'-gccacacctacctgcgagtgttcc	249

ATm5-	Reverse	5'-gacgctcctgagcacgatccaac	

MIR	Forward	5'-ttccacccactcgagcgtcaaca	158

MIR	Reverse	5'-gctcggatatgcgtgcaaccttga	

cTnT	Forward	5'-ccaagggcttcaccgggctcaa	173

cTnT	Reverse	5'-tggcagaggtggaatggatcacagg	

α-syntrophin	Forward	5'-ggactctccaccgcctccctctc	208

α-syntrophin	Reverse	5'-ccccgcttcatccttcgctctga	

β-syntrophin	Forward	5'-gacttggcttggtgggctgaacg	243

β-syntrophin	Reverse	5'-aggccattcagttgcagggtgct	

Msx1	Forward	5'-tgtacgccgctcacatgggctac	220

Msx1	Reverse	5'-ccctcttcctgccaccgaatcac	

Msx2	Forward	5'-ggaagggaagggaaggcagaacg	215

Msx2	Reverse	5'-tccacgaatcagggcgttgtttg	

β-actin	Forward	5'-tccatgaaggctgcccaact	210

β-actin	Reverse	5'-tggcgccacatctgattgat	

### Cloning of exon 1b sequence for axolotl alpha-TM

We have cloned exon 1b of the axolotl alpha-TM. 5'RACE experiments using primers designed from the exon 3 sequence of the axolotl alpha-TM (ATmC1-exon3-rev-1: 5'-CCAGACGCTCCTGAGCACGATCC; ATmC1-exon3-rev-2:5'-CCAGCTGGATACGCCTGT TC) and a 5' adaptor sequence revealed both PCR bands corresponding to the ATmC-1 sequence and a shorter PCR band after agarose gel electrophoresis. The shorter band was cloned into a pGEM-T easy vector (Promega, WI) and sequenced. The sequence shows high homology to the 5' ends of alpha-TM5a or 5b from other vertebrates. The sequence is: 5'-GTACTGTTGAGGCATCCACGTCTTCACTATTACTGGGGCATTTGTAGTCCCTTGGAATTTGAG CTGACCTTATCGCTACTCGCCTCATATGATAGAGGCGCCACACCTACCTGCGAGTGTTCCGTT CCCCGGCCCTCAGC**ATG**TCTGGGGGCACCTCCCTGGAAGCGGTGCGGCGGAAGATCCGCGC CCTGCAGGAGCAGGCGGACTCCGCTGAAGCCCGGGCGTGTAGCCTGCAGCGGGAACGAGAC GCTGAGCGGCAGCTGCGAGAGGCGGCTGAGAGTGATGTAGCCTCCCTGAACAGGCGTATCCA GCTGGTTGAGGAAGAGTTGGAT CGTGCTCAGGAGCGTCTGG. The bolded ATG represents a putative translation start site. The cloned sequence corresponding to exon 3 shows 100% identity to alpha-TM but with variations to axolotl ATmC-3, demonstrating the origin of this fragment from axolotl alpha-TM. We have named this isoform ATm5.

### Two-dimensional gel electrophoresis and Western blotting

#### Isoelectrical focusing

The immobilized pH gradient (IPG) gel strips (pH range 3-6, 11 cm, Bio-Rad, CA) were rehydrated in 200 μl ReadyPrep Rehydration/Sample Buffer that contained heart samples for 12 hours. Isoelectric focusing (IEF) was performed on the rehydrated IPGs at 20°C in three steps: 250 volts for 20 minutes, 8,000 volts for 2.5 hours, and then 20,000 Volts/hour using Protean IEF Cell system (Bio-Rad, CA).

#### Two-dimensional SDS-PAGE

After isoelectrical focusing was completed, IPG strips were prepared for SDS-PAGE using 12% Bis-Tris precast gels (Bio-Rad, CA). The strips were then rinsed with electrophoresis running buffer (pH7.7) and laid into the two-dimensional well of 12% precast gels and were run at 200 V for 1 hour following the manufacturer's protocol (ReadyPrep™ 2-D Starter Kit; Bio-Rad, CA.).

#### Western blotting

The two-dimensional SDS-PAGE gels were transferred to nitrocellulose membranes and processed for hybridization. The membranes were incubated with CH1 monoclonal antibody (1:5000) (from Developmental Studies Hybridoma Bank, University of Iowa) at a 1:2000 dilution for 1 hour at RT. After two washings for 5 minutes each, they were hybridized with horseradish peroxidase-conjugated mouse antihuman IgG (Amersham-Pharmacia Biosciences) at 1:5000 dilution for 1 hour at RT and the blots were washed and exposed to X-ray film and developed with an ECL chemiluminescence system [22].

## Results

### Cloning the full length cDNA of the myofibril-inducing RNA (MIR) gene

We have previously published the nucleotide sequence of the 166 nt long bioactive MIR [11] and showed that the artificially synthesized 166 nt RNA can promote myofibril formation and initiate beating of the mutant hearts in organ culture studies [11].

**Figure 1:**
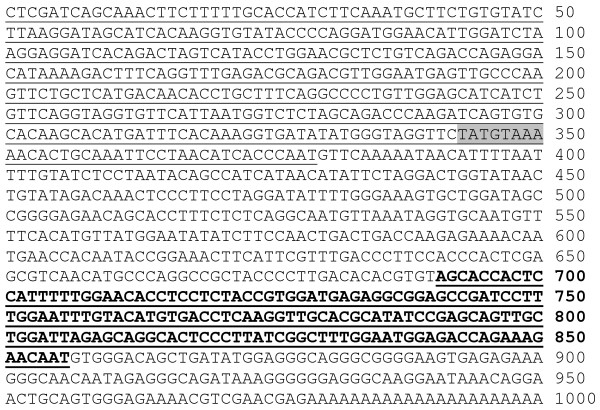
**The full length sequence of the MIR gene**. Genomic DNA sequence: 1-379 bp (Underlined); Active MIR sequence (166 nt): 691-856 bp (Bold and underlined); Potential polyadenylation signals: 835-840 bp and 840-845 bp; Predicted TATA box: 343-350 bp (Shaded); Longest predicted open reading frame: 659-808 bp; Poly A+: 977-1000 bp.

To determine the full length sequence of the MIR gene, we carried out experiments using a cDNA lambda phage library from axolotls at stage 15-17 as well as an axolotl genomic library (Stratagene, TX). Primers were designed, based both on vector flanking sequences and the 166 bp known fragment of the MIR. The PCR products were confirmed by Southern blotting assays. The promising PCR bands were then sub-cloned into pGEM-T vectors (Promega, WI) and subsequently sequenced. Primers were designed again based on the new sequence and PCRs were repeated (Table 1). By using genomic walking we have extended the 5'-end of the original 166 bp MIR to ~700 bp. Starting from the original 166 bp MIR known sequence, we also have performed 5' RACE reactions using a Smart RACE Kit (Ambion, TX). Results show that the full length of the MIR is expressed beginning at G (380^th ^base) within the genomic sequence (Fig. 1). In addition, 3' RACE reactions have revealed about 150 nt poly-A tail attached at the 3' end of the MIR (Fig. 1). We believe that we have successfully cloned the full length MIR. The nucleotide sequence of MIR has been determined and found to be unique because there is no significant homology with other known sequences available from the gene databases. Moreover, there is no relatively large open reading frame in the full-length sequence, indicating that the MIR may be functioning directly through its RNA structure, rather than a translation product. Interestingly, eukaryotic promoter prediction software (ExPASy: http://ca.expasy.org/tools/) has localized a potential promoter in the 5' genomic sequence with a conserved TATA box, 28 bp from the transcription start site (Fig. 1). Our recent data using the Luciferase reporter gene system (Promega, WI) have verified the transcription driving ability of this potential promoter. We have cloned nearly 3 kb of the upstream regulatory sequence in addition to the promoter sequence shown in Fig. 1. Serial deletions of the promoter have been inserted into the Luciferase reporter vectors and expression of these promoters has been confirmed by transfecting into rat neonatal cardiomyocytes in culture (our unpublished data).

### Full length MIR promotes tropomyosin protein expression in cardiac nonfunction mutant embryonic hearts

**Figure 2:**
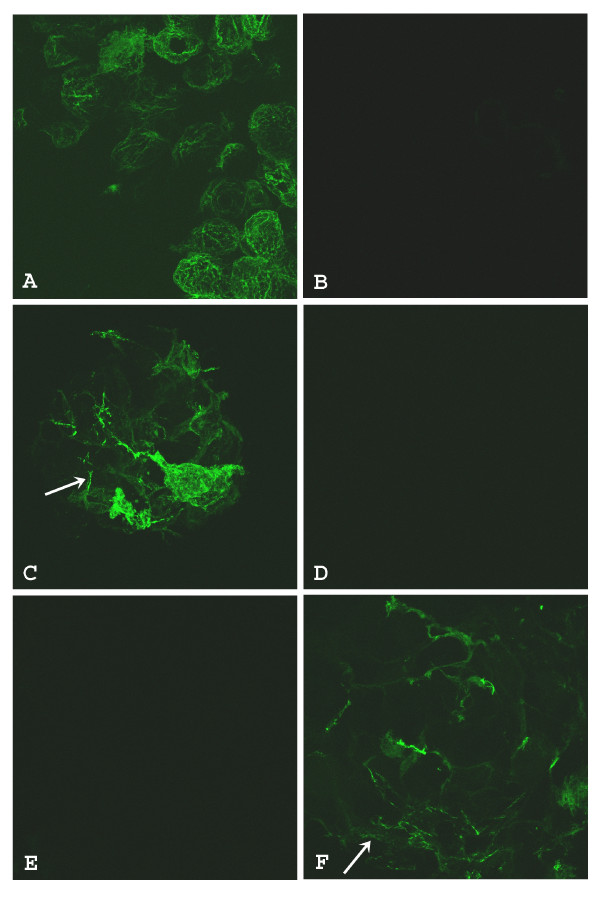
**Confocal microscopy of embryonic axolotl hearts stained with anti-tropomyosin antibody**. Normal heart incubated with Steinberg's solution and lipofectin only has extensive tropomyosin staining (A). Mutant heart incubated with Steinberg's solution and lipofectin only has virtually no staining (B). Mutant heart incubated with Steinberg's solution, lipofectin and full length sense MIR shows significant tropomyosin (arrow) staining (C). Mutant heart incubated with Steinberg's solution, lipofectin and full length sense MIR pretreated with RNase. Very little tropomyosin is indicated in the cells (D). Mutant heart incubated with Steinberg's solution, lipofectin and full length antisense MIR. No staining for tropomyosin is observed (E). Mutant heart incubated with Steinberg's solution, lipofectin and 166 nt MIR RNA shows significant levels of tropomyosin (arrow) protein (F); Magnification: 60 ×.

In vitro synthesized sense RNA from the full length (570 nt) MIR gene showed significant rescuing ability of the mutant hearts (Fig. 2). Three days after sense MIR transfection, all of the 40 dissected mutant hearts showed positive tropomyosin staining (Fig. 2C) while antisense RNA transfection showed only background levels of staining (Fig. 2D). Pretreatment of the MIR sense RNA with RNase before transfection into the mutant hearts totally abolished the activity of the sense RNA in promoting tropomyosin expression (Fig. 2E). It is clear that the bioactivity of promoting tropomyosin expression in the mutant hearts is due specifically to the sense MIR. These results are in total agreement with our previous findings using the short version of the MIR (166 nt). The full length 570 nt RNA used for transfection in current experiments is at the same molar concentration as the 166 nt RNA we used for previous studies [7,11]. Since the original 166 nt RNA (partial sequence from MIR gene) is sufficient for the rescuing of mutant hearts (Fig. 2F) [7,11], we believe that the 166 nt partial sequence probably is the functional bioactive unit in the MIR gene.

### MIR-promoted tropomyosin protein expression in the mutant hearts is not due to increased transcription levels or splicing pattern changes of tropomyosin genes

To determine whether MIR-promoted tropomyosin protein expression in the mutant heart is due to regulation at the transcriptional levels or changes in the splicing patterns of the TM transcripts, we proposed two hypotheses. We tested those hypotheses by performing a series of real-time RT-PCR experiments.

**Figure 3:**
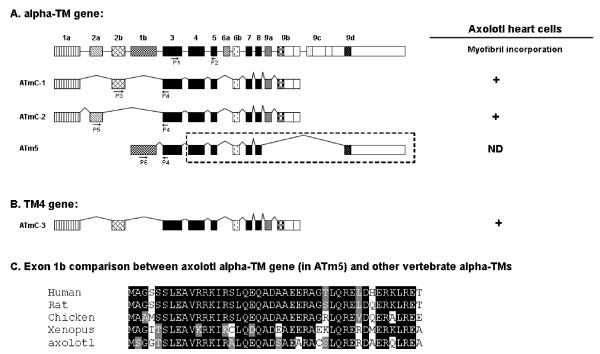
**Schematic representation of four different TM isoforms produced from alpha -TM (A) and TM4 type tropomyosin (B) genes**. Each box represents an exon which is joined together by mRNA splicing. The alpha-TM gene is alternatively processed at both the 5' and/or 3' ends, as well as at the internal exons 6a/b. Our studies are focused on the 5' end sequence and exon 2a/b. The fibroblast type tropomyosin isoform (ATm5) has only the 5' sequence cloned with exon 1b and part of exon 3 cloned for RT-PCR studies, assuming the internal and 3' sequence are homologous to other vertebrates (dashed line box). Primers to amplify specific isoforms were designed based on the alternative spliced exons (P1 to P5). C. Highly conserved peptide sequence from exon 1b of axolotl alpha-tropomyosin compared to other vertebrate sequences. The indicated splicing pattern of axolotl tropomyosin genes are hypothetical based on other vertebrate sequences. ATmC-1, 2 and 3 were all demonstrated to be able to incorporate into myofibril structures [21].

**Figure 4:**
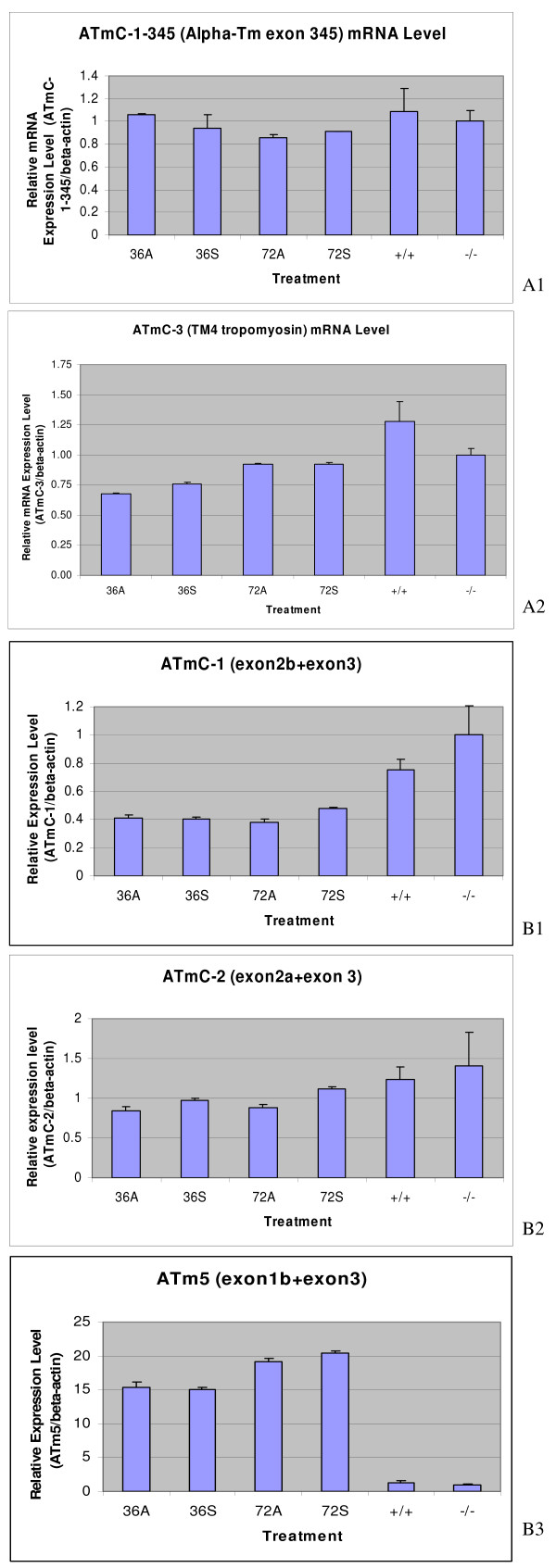
**Transcriptional levels of various tropomyosin isoforms (A1 and A2); Determination of mRNA splicing of various tropomyosin isoforms (B1, B2 and B3)**. Real-time RT-PCR showed no significant difference at the transcription level for alpha- (A1) and TM4 type tropomyosin (A2) genes in the mutant hearts treated with MIR sense or antisense RNA. 36A: Mutant hearts treated with antisense MIR for 36 hours; 36S: Mutant hearts treated with sense MIR for 36 hours; 72A: Mutant hearts treated with antisense MIR for 72 hours; 72S: Mutant hearts treated with sense MIR for 72 hours; +/+: homozygous normal hearts with no treatment; (-/-): homozygous mutant hearts with no treatment. Real-time RT-PCR showing no significant difference during mRNA splicing of the alpha-TM gene (the ATmC-1 containing exon 2b (B1), the ATmC-2 containing exon 2a (B2) and the fibroblast tropomyosin isoform with direct exon 1b and 3 conjunction (B3) in the mutant hearts treated with the MIR sense or MIR antisense.

Our first hypothesis proposes that for the MIR to promote tropomyosin protein expression, it must increase the expression of one or both of the tropomyosin genes (alpha-tropomyosin and TM4 type) at the transcription level. For the real-time RT-PCR experiments, we designed primer pairs to amplify the alpha-tropomyosin and TM4 type tropomyosin genes selected from the common exon sequences whose products represent the total transcripts of each gene (Table 1). For alpha-TM, primers (ATmC-345-for and ATmC-345-rev) are designed in the Exon 3, 4 and 5 regions which are common to all alpha-TM transcripts [24,25]; for TM4 type tropomyosin, primers (ATmC-3-for and ATmC-3-rev) are designed in the 3' UTR region (Fig. 3A, B). Our real-time PCR results clearly show that there is no significant difference between alpha-tropomyosin or TM4 type tropomyosin RNA transcripts in normal (+/+), mutant (c/c), sense MIR- and antisense MIR- treated mutant embryonic hearts (stage 37-38) at two different time periods of the transfection of MIR (Fig. 4A1, A2). These results clearly demonstrate that the MIR does not perform a regulatory function at the transcriptional level, thus disproving the first hypothesis.

Our second hypothesis proposes that the MIR regulates splicing of tropomyosin transcripts to produce correct isoforms in the cardiac muscle cells. For these real time RT-PCR studies, we designed specific primer pairs based on representative alternative spliced exons of the alpha-tropomyosin gene. From our previous studies, we found that both ATmC-1 and ATmC-2 cDNAs are expressed from the same alpha-tropomyosin gene but differ in exon 2, with ATmC-1 containing a striated muscle specific exon 2b while ATmC-2 contains a smooth muscle isoform specific exon 2a (Fig. 3A). We have shown previously that both ATmC-1 and ATmC-2 can be incorporated into cardiac muscle fibers [21]. Alpha-TM has been reported also to produce fibroblast tropomyosin isoforms with smaller molecular weights, such as TM5a and TM5b, both of which can be incorporated into myofibrils [25]. Isoforms TM5a and 5b are produced by using an internal promoter located between exon 2b and exon 1b thus skipping exon 2a and exon 2b and conjoining exon 1b and exon 3 directly together [26]. To determine whether MIR can regulate the expression pattern of tropomyosin by selectively favoring the use of exon 1b, 2a or 2b, we have cloned exon 1b of the axolotl alpha-TM to study the Axolotl Tm5 gene (ATm5). 5' RACE experiments using primers designed from the exon 3 sequence of axolotl alpha-TM and a 5' adaptor sequence revealed PCR bands corresponding to the ATmC-1 sequence and a shorter PCR band by agarose gel electrophoresis. The shorter PCR band was gel purified and cloned into T-vectors (Promega, WI). Sequencing results demonstrated a highly conserved sequence of axolotl alpha-TM exon 1 compared to other vertebrates (Fig. 3C). We then designed different primer pairs based on the alpha-TM exon 1b, 2a and 2b sequences (Fig. 3A). Real-time RT-PCR was performed on normal, mutant, MIR sense and antisense RNA-treated mutant embryonic hearts using these primer pairs to compare alpha-TM splicing patterns. Results from these experiments did not support our second hypothesis. That is, the alpha-TM splicing pattern at the 5' end is not regulated by the MIR. Neither ATmC-1 (exon 2b included) nor ATmC-2 (exon 2a included) type splicing is altered with MIR treatment (Fig. 4B1, B2). Similar quantities of splicing products also were observed for fibroblast type tropomyosin with direct exons 1b and 3 conjunctions in all normal, mutant, MIR sense and antisense RNA treated hearts (Fig. 4B3).

Thus these studies reiterate that MIR-promoted tropomyosin expression in the mutant hearts is not due to increased transcription levels or splicing pattern changes in the tropomyosin genes.

### Knockdown of MIR expression decreases sarcomeric tropomyosin protein resulting in the failure of myofibril formation in normal hearts

**Figure 5:**
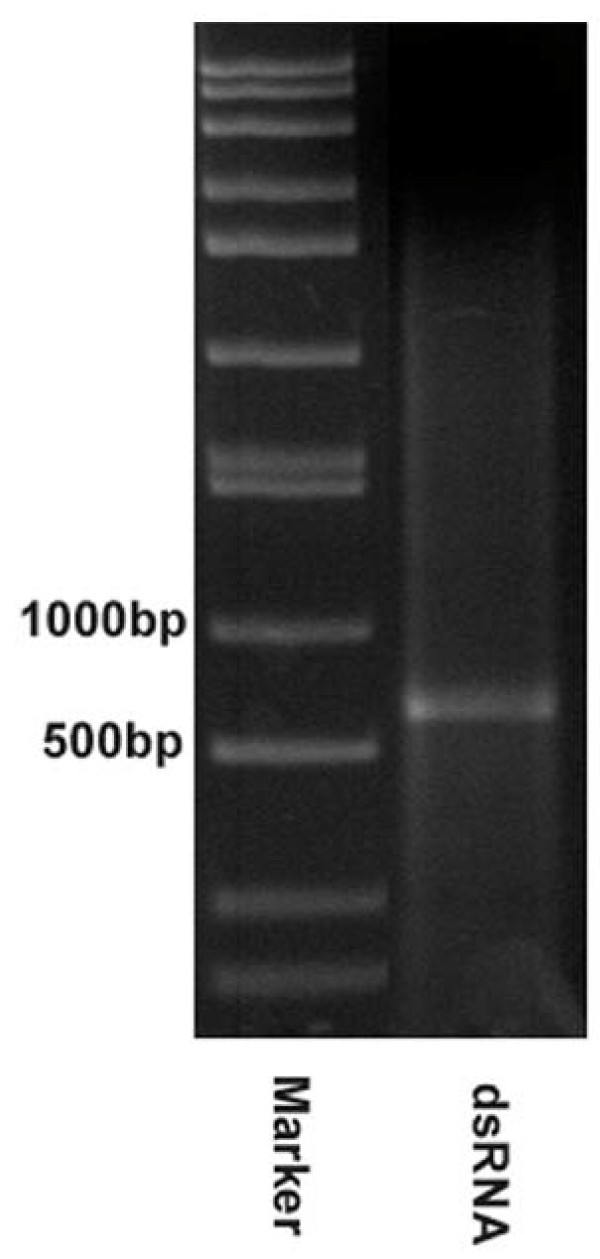
**Double-stranded MIR**. Synthesized MIR double-stranded RNA loaded on 1.5% agarose gel for electrophoresis shows a 550 bp band. The size marker is loaded on the left-side of the gel.

**Figure 6:**
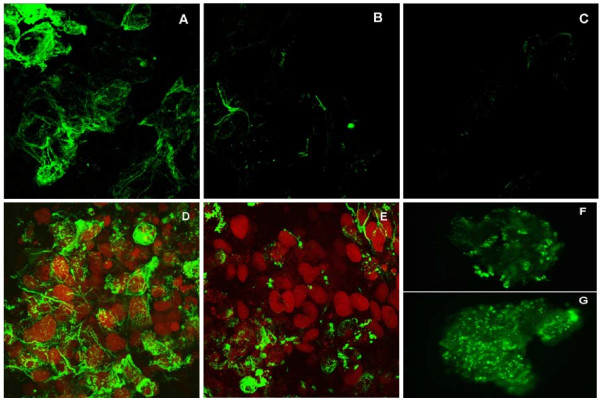
**Confocal microscopy of embryonic axolotl hearts treated with double-stranded MIR**. The images show those hearts that stopped beating after 4 days but regained contractions later. Hearts were fixed after 9 days in culture. Normal heart (stage 36) organ cultured with lipofectin in Steinberg's Solution only for 10 days (A); Myofibril structures are clearly shown. Normal heart (stage 36) organ cultured with lipofectin in Steinberg's Solution with double-stranded RNA for 10 days (B); Mutant heart (stage 36) organ cultured with lipofectin in Steinberg's Solution with double-stranded RNA for 10 days (C). (Magnification: A-E: 60 ×; F and G: 10 ×). Hearts incubated with mock transfection (no dsRNA) show abundant staining of tropomyosin and organized myofibril structures (D) with nuclei stained by propidium iodide (PI). Hearts transfected by the dsRNA show a significant decrease of tropomyosin expression in more than half of the cells but continue to express in the remaining cells (E). Normal hearts treated with dsRNA for 9 days stained for tropomyosin (F). Clusters of cells with positive staining for tropomyosin are present in different areas. Normal hearts with mock transfection (no dsRNA) for 9 days stained for tropomyosin (G).

Our confocal immunofluorescence studies have proved that MIR is sufficient to promote tropomyosin expression and myofibrillogenesis in cardiac mutant hearts. To demonstrate that the MIR is essential for normal embryonic heart development in axolotls, double-stranded MIR (550 bp covering the full length) was synthesized as described in the Materials and Methods section. Successful production of double-stranded MIR (dsRNA) has been verified by polyacrylamide gel electrophoresis (Fig. 5). Using the same methods, we have also produced FITC-labeled dsRNA. The high transfection efficiency using these dsRNAs was demonstrated by confocal microscopy. It showed nearly 100% of the heart cells in the embryonic heart tube were transfected using our transfection protocol. The double-stranded RNA was purified from single stranded RNA residue by agarose gel electrophoresis and was applied to the cultured normal whole embryonic hearts. We found that this RNA was effective in inhibiting the normal heartbeat as determined by daily evaluation under a dissecting microscope (Table 2). Monoclonal antibody staining (CH1) for tropomyosin [7,27] in these double-stranded RNA-treated hearts revealed disruption of myofibril formation (Fig. 6).

**Table 2:** Percentage of beating hearts at different time points after ds RNA transfection.

Treatment Groups	Day 0 (0 hr)	Day 4 (96 hrs)	Day 9 (216 hrs)
dsRNA treated group	100%	26.67%	46.67%

RNase-pretreated dsRNA treated group	100%	76.67%	70%

Steinberg's solution only group	100%	96.67%	76.67%

The dissected normal hearts with mock transfection (no RNA) showed abundant tropomyosin expression and organized myofibrils easily detectable by confocal microscopy even after 9 days in culture (Fig. 6A). Most double-stranded MIR-transfected hearts (22 out of 30 total hearts at stage 37) when dissected stopped beating after 4 days in culture. Careful observation of these hearts under an inverted microscope (× 200) revealed sporadic contractions in portions of the heart tube indicating that these hearts have live tissue. We evaluated the viability of the cells in the organ-cultured hearts by staining the whole hearts with Trypan Blue. Interestingly, 9 out of the 22 ds MIR-transfected hearts that had stopped beating, resumed contraction activity after 6 days in culture although at a decreased beating rate and strength, while the rest remained quiescent for the duration of the experiment (9 days). Confocal microscopy on these hearts that stopped beating and never regained their contractility after 9 days in culture showed a significant disarray in the myofibril structures and decreased levels of tropomyosin protein expression (Fig. 6B), similar to the untreated mutant hearts [7,21]. No rescuing effect was observed in the mutant hearts transfected by ds RNA and also they showed a negative staining pattern for tropomyosin expression (Fig. 6C). A mock-transfected (no RNA) normal heart showed numerous cells positively stained by tropomyosin antibody, CH-1 (Fig. 6D). Fig. 6E shows a normal heart treated with double-stranded MIR. It clearly showed significantly lower tropomyosin expression than normal levels in the whole heart tube except for the conus region, which showed a few positively stained cells. Results are reminiscent of a similar staining pattern for mutant hearts in our previously published study [21], with negative staining for tropomyosin in the ventricle but positive staining in the conus region. Since ds RNA could be degraded by Nuclease S1 [28], we also included a control group of normal hearts transfected with dsRNA predigested with Nuclease S1 (Promega, WI) in our experiments, to determine whether degraded double-stranded RNA has any effect on normal heart beating. Interestingly, after transfection with the degraded double-stranded RNA, these hearts stopped beating temporarily (possibly due to the damaging effects of RNases remaining in the medium) but regained normal beating activity after an additional 3 days in culture. Confocal microscopy showed numerous positively-stained cells in these hearts which appeared similar to untreated normal hearts after 9 days in culture.

In the double-stranded MIR RNA treated normal hearts that stopped beating after 3 or 4 days but regained contractions after 9 days, confocal microscopy studies still revealed decreased staining of tropomyosin protein (Fig. 6F) compared to mock transfected cells (Fig. 6G). Organized myofibrils with tropomyosin staining could be found in a few sparsely distributed cells in the heart although the overall numbers of stained cells were dramatically decreased in the dsRNA-treated hearts (Fig. 6E) compared to the control normal hearts without dsRNA treatment (Fig. 6D). Since our whole-mount heart specimens are usually 150-200 μm in thickness, we used epifluorescence microscopy to view the fluorescence image of the whole heart. Thus we were able to clearly observe the groups of cells that show positive staining in the dsRNA-treated whole hearts (Fig. 6F, G).

**Figure 7:**
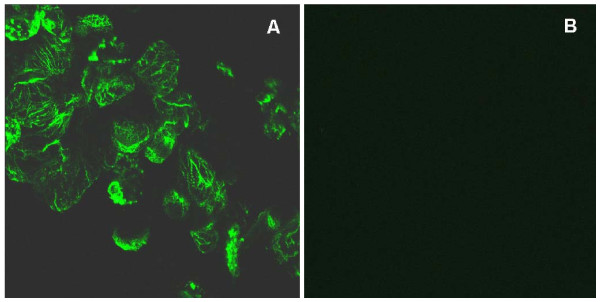
**Myofibril morphological examinations**. Normal embryonic hearts treated with double-stranded MIR for 4 days show a dramatic reduction in tropomyosin protein and myofibrils. Hearts scanned here with dsRNA transfection already have stopped beating at the time of fixation. Hearts incubated with mock transfection (no dsRNA) show abundant staining of tropomyosin and organized myofibril structures (A). Hearts transfected by dsRNA show almost no detectable expression of tropomyosin in the whole area of the ventricle (B). (Magnification: 60 ×).

Immunofluorescence confocal microscopy also has showed the decrease in tropomyosin expression in normal hearts that stopped beating after transfection with double-stranded MIR for 4 days. In some of the hearts, the loss of cells with positive tropomyosin protein staining and organized myofibrils structure is nearly 100% in the ventricular area of the heart tube (Fig. 7A, B). We observed some cells in a random pattern that have positive green signal from tropomyosin staining, but with a much lower signal intensity. In hearts that stopped beating after 4 days of transfection with double-stranded MIR, their tropomyosin staining was much weaker in general and there were far fewer cells with positive staining for tropomyosin as compared to the hearts from day 9. The high percentage of cells with decreased tropomyosin expression in the whole heart tube after double-stranded MIR transfection is due to our high efficiency of transfection of double-stranded MIR into the heart cells, as described in the Materials and Methods section. The efficiency in knocking-down tropomyosin expression as well as visualization of its differential expression was likely due to direct exposure of cardiomyocytes to the MIR in solution because of an absence of the epicardial layer in hearts at stage 37/38 [29].

These findings strongly suggest that the MIR gene is essential for myofibrillogenesis and heart development in normal embryonic hearts.

### The transcription levels of ATmC-1 and 2 (alpha-TM) and ATmC-3 (TM4) genes are not altered by short term but decrease after long term MIR knockdown following double-stranded RNA treatment

**Figure 8:**
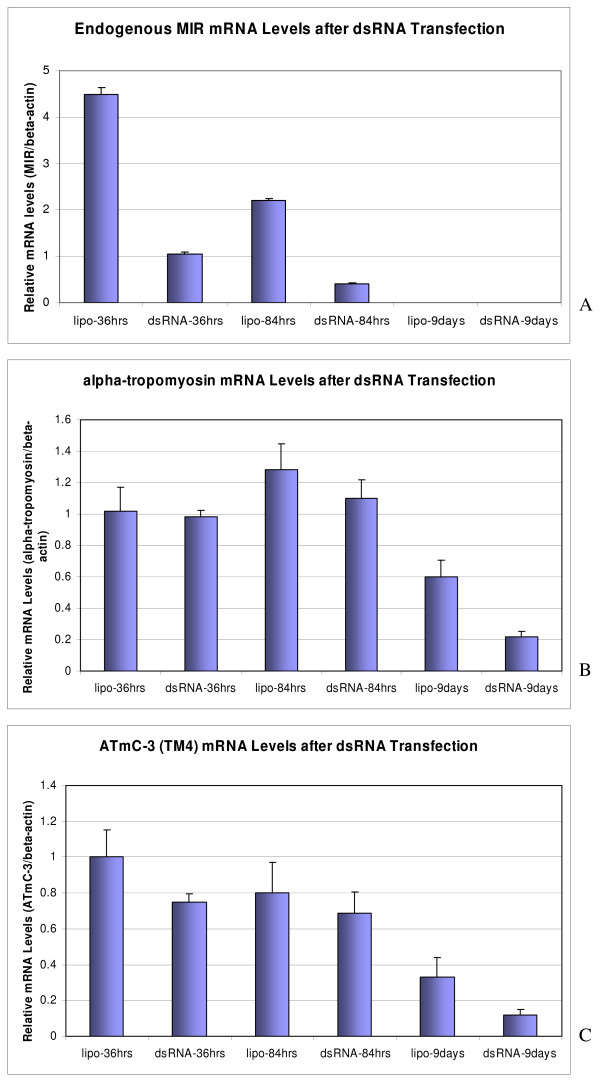
**Determination of myofibril protein expression**. Real-time RT-PCR confirms the knockdown of endogenous MIR (A) in the normal hearts but shows no significant difference at the level of the transcripts of alpha- (B) and TM4 type tropomyosin (C) transcripts in the normal hearts treated with double-stranded MIR (dsRNA-) for 36 hours or 84 hours. However, both alpha- and TM4 type tropomyosin transcripts in the normal hearts treated with dsRNA for 9 days show dramatic decreases to nearly 1/3 of the levels in control groups with hearts incubated in lipofectamine transfection medium only. MIR expression in 9-day-cultured hearts is not detectable.

On treatment with ds-RNA, we observed that a significant number of hearts stopped beating at days 3 and 4. To determine the transcriptional levels of tropomyosin genes following ds-RNA treatment, we collected the total RNA from hearts treated with dsRNA and the negative controls (cultured in Lipofectin/Steinberg's solution only, with no dsRNA) after a 2-day transfection period with dsRNA (24 to 48 hours before significant decreases of tropomyosin protein were observed). Gene expression levels were then analyzed for both alpha-tropomyosin and TM4 type tropomyosin as well as the endogenous MIR. We observed that the endogenous expression of MIR was knocked down about 80% in normal hearts 36 or 84 hours after dsRNA transfection compared to the negative controls (Fig. 8A). It should be noted that a gradual decrease of MIR expression in untreated hearts was observed relative to in vitro culture times which is consistent with our earlier reports that MIR expression decreases progressively during in vivo development [7]. Two of the major isoforms of tropomyosin expressed in axolotl hearts alpha-tropomyosin (ATmC-1 and ATmC-2) and TM4 type tropomyosin (ATmC-3), interestingly, were not significantly reduced at the transcriptional level after a short period of treatment with double-stranded MIR (Fig. 8).

However, after collecting RNA samples from hearts organ-cultured for 9 days with either dsRNA or the no RNA controls, we found that both the alpha-tropomyosin and TM4 type tropomyosin decreased in mRNA levels in our real-time RT-PCR assays (Fig. 8). No significant changes in the tropomyosin expression levels were observed in dsRNA-treated hearts compared to the mock-transfected normal hearts (no dsRNA) in alpha-tropomyosin (ATmC-1 and ATmC-2) and TM4 type tropomyosin total RNA transcripts at either 36 hours or 84 hours post-transcription. A confirmation of the effect of double-stranded RNA transfection was shown by down regulation of the endogenous MIR to 1/4^th ^(36 hours) to 1/5^th ^(84 hours) dsRNA transfection (Fig. 8A). Decreased expression levels for both types of tropomyosin have also been detected in the 9-day-cultured hearts compared to the hearts cultured for only 36 or 84 hours.

These results indicate that MIR possibly influences tropomyosin gene expression by an indirect regulatory mechanism. We also showed that decreasing the tropomyosin protein level in normal hearts after double-stranded MIR treatment is not due to changes of alpha-tropomyosin (ATmC-1 and 2) or TM4 tropomyosin (ATmC-3) mRNAs, but rather due to a failed translational or posttranslational control.

### The reduced expression of tropomyosin may be related to the differential regulation of MIR of various tropomyosin isoforms in normal and mutant hearts

**Figure 9:**
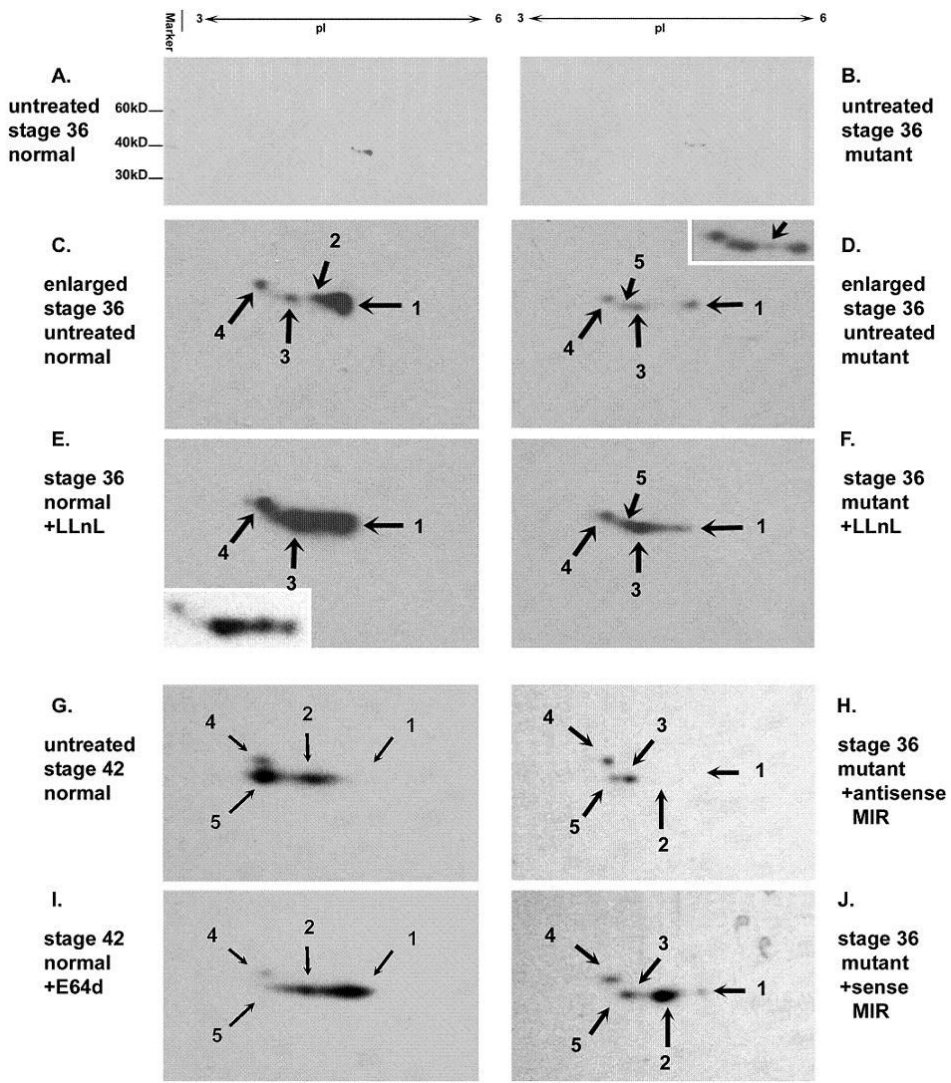
**Two-dimensional SDS-PAGE of embryonic heart proteins followed by Western Blotting using CH1 (anti-tropomyosin) antibody**. Untreated stage 36 normal hearts (A); Untreated stage 36 mutant hearts (B); Enlarged figure from A for CH1-recognizable tropomyosin isoforms in untreated normal hearts (C); Enlarged figure from B for CH1-recognizable tropomyosin isoforms in untreated normal hearts (D); The top right corner after overexposure of the same blot as D shows that mutant hearts are expressing isoform 2 as well as isoforms 1, 3 and 4. Mutant hearts at this stage are expressing all of the isoforms as normal hearts at much lower levels along with an extra isoform (5) which is detectable only at later developmental stages in normal hearts (G); Stage 36 normal hearts incubated with LLnL for 10 hours. The left bottom corner shows the less exposed image after Western blotting on the same sample, clearly showing increased protein concentration for isoform 2, 3 and 4 but not for 1 (E); Stage 36 mutant hearts incubated with LLnL for 10 hours. Increasing of protein concentration for isoform 3, 4 and 5 is clearly detected but not for 1 (F); Untreated stage 42 normal hearts (G); Stage 42 normal hearts incubated with E64d for 10 hours (I); Protection for isoform 1 is detected. Stage 36 mutant hearts treated by antisense MIR for 4 days (H); Stage 36 mutant hearts treated by sense MIR for 4 days (J); A significant increase in spot density for isoform 2 is detected when compared to untreated mutant hearts at the same stage (D).

With the observation of normal control of tropomyosin expression at the mRNA level in mutant hearts, we have extended our studies of tropomyosin expression at the protein level. To determine whether multiple isoforms of tropomyosin exist in embryonic axolotl hearts and to verify if they are differentially expressed in mutant hearts, we used two-dimensional Western blotting experiments. Using a monoclonal antibody (CH1), developed to recognize sarcomeric type tropomyosin specifically [30], we have detected five different protein spots (tropomyosin isoforms) from both normal and mutant embryonic hearts at stages 36 to 42 (Fig. 9). All isoforms of tropomyosin detected by the CH1 antibody are located between pI 4 to 5 with molecular weight of approximately 38 kD. As shown in the Fig. 9C, D, we have found that, the protein levels of all the isoforms are significantly decreased in the mutant hearts compared to the normals. The No. 2 protein spot is barely shown in the enlarged blot but we can clearly see this spot in the overexposed film as shown in the right top corner (inset) of Fig. 9D. With reference to protein spot number 5, it was not detectable in either normal or mutant heart samples at stage 36 (initiation of normal heartbeat stage). However, at stage 42, the number 5 protein spot was prominent in normal heart samples but nonexistent in mutant hearts.

As shown in Fig. 9, we have observed an isoform switch between embryonic hearts at stage 36 (Fig. 9D) and stage 42 (Fig. 9G). The stage 36 hearts predominantly express isoform 1 while the stage 42 hearts express isoforms 2 and 5. To further confirm the active translational activity for tropomyosin proteins in mutant hearts, we have treated the mutant hearts with the cysteine-protease inhibitor, N-acetyl-leucine-leucine-norleucinal (LLnL, Calbiochem, San Diego, CA). The LLnL is a membrane-permeable proteinase inhibitor that inhibits Calpain and proteosome and has been reported to affect tropomyosin turnover in other cell culture systems [31]. All tropomyosin isoforms, except for No. 1, show significant increases in both normal and mutant hearts after incubating with LLnL for 10 hours. Using another lyosomal cysteine proteinase inhibitor, E-64d (Sigma, St. Louis, WA), which inhibits Calpain but not proteosome, interestingly showed increased expression of all isoforms including No.1 (Fig. 9I). These results suggest that different tropomyosin isoforms are degraded by different proteinases.

To confirm the increased expression level of tropomyosin protein after sense MIR treatment of mutant hearts, we conducted 2D Western blotting experiments using protein samples from mutant hearts treated with sense or antisense MIR for 4 days. (Fig. 9D, H and 9J), show that antisense MIR treatment decreases the amount of tropomyosin isoform 1 in mutant hearts without altering isoforms 3, 4 and 5. A significant increase of isoform 2, one of the major isoforms of tropomyosin in the embryonic heart at stage 42, is also detected. However, an unexpected loss of isoform 5 is observed in the sense MIR-treated mutant heart sample. These results unequivocally prove that MIR post-transcriptionally controls tropomyosin expression in embryonic hearts. In normal and mutant heart samples, irrespective of stage or MIR treatment, isoform 4 is always detectable with negligible alteration in expression, indicating that it probably has a housekeeping function.

### MIR promotes expression of myofibril structural protein genes

**Figure 10:**
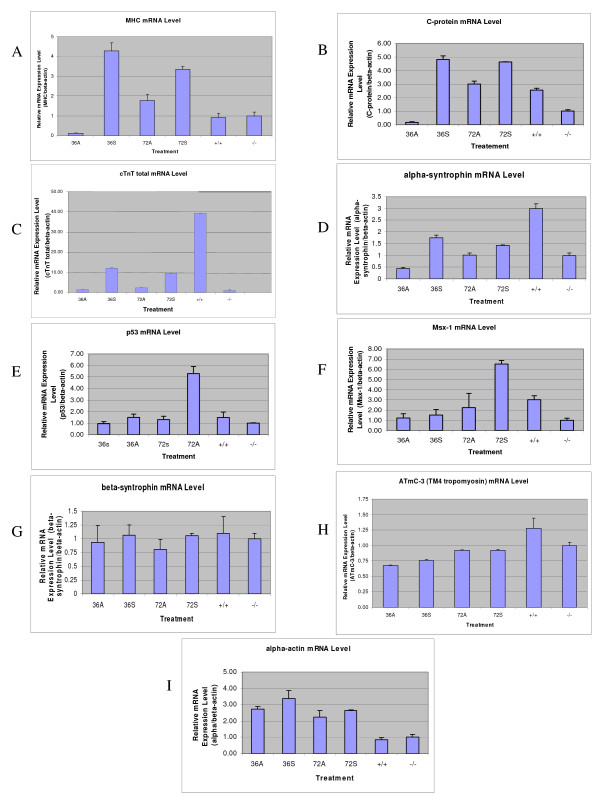
**Real-time RT-PCR of myofibril genes on total RNA from stage 35-37 embryonic hearts treated by MIR**. Relative expression levels of different genes were compared after normalizing by beta-actin mRNA levels. 36A: antisense MIR treated for 36 hours; 36S: sense MIR treated for 36 hours; 72A: antisense MIR treated for 72 hours; 72S: sense MIR treated for 72 hours; -/-: homozygous mutant hearts with no treatment; +/+: homozygous normal hearts with no treatment. Alpha-MHC (A), C-protein (B), cardiac troponin T (C), alpha-1-syntrophin (D), p53 (E), Msx-1 (F), beta-1-syntrophin (G), ATmC-3 (H), cardiac actin (I).

From the previous experiments it is clear that MIR has a significant influence on the expression of tropomyosin in the developing heart which is known to play a major role in mechanically stabilizing actin filaments [32]. In this investigation we extended our gene expression studies to determine whether MIR promotes the expression of myofibril structural genes other than tropomyosin, such as, α-myosin heavy chain, myosin-binding C-protein and cardiac troponin T and α-syntrophin (one of the dystrophin-associated proteins), in normal and mutant hearts (Fig. 10). For the real-time RT-PCR experiments, primer pairs for each gene were designed in the intron-flanking exon sequences to avoid PCR amplifications from genomic DNA contaminants. A significant increase in the mRNA of α-MHC, C-protein, cTnT and α-syntrophin were observed after 36 hours of incubation of mutant hearts (stage 37-38) with sense MIR, but not with antisense RNA. However, the effects of MIR treatment diminished after 72 hours, indicating possible degradation of MIR in the heart cells (Fig. 10G). On the other hand, p53 and Msx-1 genes, both being cell differentiation-related genes, show increased expression in the mutant hearts after 72 hours of MIR treatment, indicating an indirect or long term effect of MIR on promoting embryonic cardiomyocyte differentiation.

In summary, there are three categories of myofibril structural genes that respond to the treatment of MIR differently at their mRNA level: 1) genes that can be quickly (36 hours) up-regulated in transcription by MIR sense RNA treatment (α-MHC, C-protein, cTnT and α-syntrophin); 2) genes that can be slowly (72 hours) up-regulated by MIR sense RNA treatment (p53 and Msx-1); and 3) genes that are not affected by MIR treatment (cardiac actin, β-syntrophin and ATmC-1, -2 and -3).

## Discussion

Our research on the MIR gene has been the first to demonstrate that a small RNA is involved in the regulation of embryonic heart development in the cardiac mutant (gene c) of *Ambystoma mexicanum*. In the case of the MIR gene, since we now know that the MIR is a polyadenylated RNA, there is reason to believe that it may function through its unique secondary structure to promote myofibrillogenesis in the mutant hearts. We favor this hypothesis for the following reasons: First, the RNA used for rescuing experiments (either 650 nt full length or 166 nt clone #4 RNA) was not processed for translation by capping or polyadenylation before it was added to the heart cultures implying that it lacked necessary machinery for translation inside the cells. The possibility that the active RNA rescues mutant hearts by providing mRNA that is translated is unlikely. Second, although there are several small open reading frames (ORF) predicted for the full length gene (the longest ORF is shown in Fig. 1), the shortest RNA we have shown to preserve bioactivity (140 nt with 26 nt deleted from the 3' end of 166 nt Clone #4 sequence shown with an underline in Fig. 1) does not cover the whole ORF [33]. Thus, the chance of this RNA being translated in the cells is virtually nonexistent.

Recently, there have been numerous tissue-specific and embryonic stem cell-expressed microRNAs cloned from mouse, some of which are heart specific [34,35]. Prior to our finding on the MIR [7,11] there had been no previously reported non-coding RNAs important for heart development). In fact, the MIR gene appears to be the first non-coding RNA that has been reported to be involved in the process of anterior endoderm inducing precardiac mesoderm to form functional cardiac muscle tissue [36]. Further characterization is underway to rule out the possibility of MIR as being a typical miRNA and/or consider as a piRNA [37-39] or some other unique regulatory RNA molecule.

One of the long term objectives of our studies is to find the mammalian homolog of the MIR gene and study related pathways in mammals, including human. Although some of the noncoding RNAs, such as some micro RNAs, are conserved between species, there are others that have many variations [40]. Based on sequence similarity, we have not yet identified the mammalian MIR homolog gene from a database search although we have preliminary data indicating that both sheep heart [7] and human heart (our unpublished data) contain functional MIR homologs which are able to promote myofibrillogenesis and rescue the mutant axolotl hearts. Recently, Mummery et al. [41] have indicated that there exists a very similar, probably identical, induction mechanism for mouse heart development from signals secreted from cultured mouse visceral endoderm-like cells. This factor has been found to be capable of promoting beating cardiomyocyte differentiation and beating in human embryonic stem cell cultures, indicating a non-protein factor secreted from mouse visceral endoderm cells, possibly the mouse homolog of our MIR gene. Thus, these results and findings from our studies in the animal model, *Ambystoma mexicanum*, suggest that the relationship between endoderm and heart induction is genetically conserved across the vertebrate species.

We have previously demonstrated that the MIR (166 nt core sequence) can bind to more than one protein. The MIR gene and its binding proteins are apparently involved in the regulation of tropomyosin expression [7]. Prior to current studies, it was not clear whether the failure of tropomyosin expression in mutant hearts takes place at the transcriptional level or the translational level and which isoform(s) of tropomyosin is/are significantly reduced in the mutant embryonic hearts. Our results in this study prove unequivocally that the MIR and, perhaps, its binding protein(s) work together to regulate tropomyosin expression translationally or posttranslationally in the mutant hearts since our studies did not detect transcriptional or splicing pattern differences in tropomyosin genes between normal and mutant embryonic hearts. Moreover, sequencing of the full-length cDNAs of both alpha-tropomyosin and ATmC-3 from the embryonic hearts of both normal and mutant axolotls showed no differences reiterating the possible non-existence of splice variants of these genes in mutant hearts.

In addition to its significance for studying the basic mechanisms of myofibrillogenesis, irregularities in tropomyosin expression have also been shown to have clinical significance. There have been a number of reports indicating that familial cardiomyopathy can be caused by mutations in the tropomyosin gene itself [42-44]. Homozygous alpha-TM knockout mice are embryonic lethal [45]. Therefore, the cardiac mutant axolotl, with its deficiency in tropomyosin and its ability to be corrected, is potentially a very important model system for studying regulation of tropomyosin isoforms. It is clear from our analyses of the cardiac lethal mutant axolotl, that gene '*c' *does not control early heart morphogenesis since the heart appears to form normally at pre-heartbeat stages [46]. However, the expression of tropomyosin is significantly reduced at the heartbeat stage (stage 35) [47]. Moreover, fewer isoforms of tropomyosin are detectable in embryonic mutant hearts by two-dimensional gel electrophoresis as compared to normal hearts [4]. This tropomyosin deficiency is intriguing given the current understanding of tropomyosin genetics and expression [16,17,25,44,48]. Despite these numerous studies, the mechanisms of tropomyosin gene regulation in cardiac tissues are not completely understood.

In addition to the identification of tissue specific transcription factors that regulate different tropomyosin gene transcription and specific intron/exon splicing regulatory sequences and factors that control alternative splicing, there are numerous recent findings that show both translational and posttranslational (sorting and protein turnover) control on tropomyosin proteins. For example, Rethinasamy et al. [45] have shown that translational regulation plays a major role in tropomyosin expression. In heterozygous knockout mouse hearts with a 50% reduction in cardiac muscle α-TM mRNA, no compensatory increase in transcript levels were found for striated muscle β-TM or TM-30 isoforms. However, normal amounts of striated muscle α-TM protein are produced and integrated into the myofibril, suggesting a mechanism for adjusting translational levels in functioning mouse hearts.

Besides the sorting mechanism for specific tropomyosin isoform subcellular localization, proteinase degradation is another important regulatory mechanism in the posttranslational control of tropomyosin. In non-muscle normal rat kidney (NRK) cells, it has been reported that large molecular weight tropomyosin proteins are degraded faster than smaller molecular weight tropomyosin proteins indicating different proteinases are involved in their proteolysis [31]. The stress fiber component tropomyosin degradation in normal cells or under growth factor treatment can be blocked by LLnL, a proteinase inhibitor to lysosomal cathepsins B and L, cytoplasmic calpains I and II [49] and proteasomes. However, using inhibitors that block calpains but not proteasomes does not block TM degradation [50]. These results indicate that striated muscle myofibril structural tropomyosins, stress-fiber high molecular weight tropomyosins and small molecular weight non-muscle tropomyosins are degraded by different proteinases within the cell. In our studies using axolotl heart tissue, we have also shown that LLnL can prevent degradation of some striated muscle tropomyosin proteins (CH1 antibody recognizable) but not all, especially isoform number 1 that has increased expression along with heart development but has not been protected from the degradation by LLnL. Another Proteinase inhibitor, E-64d, inhibits Calpain activities but not proteosome activites and thus protects a different subset of tropomyosin isoforms including isoform number 1 in the embryonic hearts.

In our mutant axolotl system, the decreased translational level of tropomyosin protein, abnormal tropomyosin sorting and significantly higher rates of protein degradation of the tropomyosin protein could all exist as a potential foundation for this mutant phenotype. However, recent studies on mutant hearts with normal ATmC-1, ATmC-2 and ATmC-3 tropomyosin cDNA transfected constructs, clearly show reorganization of myofibril structures, indicating the possible existence of a sorting mechanism in mutant cardiomyocytes [20,51].

Two possible mechanisms by which MIR promotes tropomyosin synthesis and myofibrillogenesis in the mutant axolotl hearts might include:

1. A mechanism whereby the MIR enters the mutant heart cells, is transported into the nucleus, perhaps together with its binding protein(s) [7], and promotes gene transcription of the various myofibril components. The MIR could as well regulate RNA splicing processes for these genes as indicated in our studies on cardiac troponin T [23]. The myofibrillogenesis is thus promoted with increased expression of building blocks of myofibrillar proteins. While the nascent myofibrillar structures may incorporate tropomyosin proteins and protect them from being degraded in MIR treated hearts, it is possible that those proteins, existing in unprotected monomer form in the untreated hearts, get degraded by unknown proteinase(s).

2. A second possibility might be that MIR and its binding proteins promote gene expression followed by translation of protein products that facilitate the translation of the mRNA of tropomyosin and possibly others, thus providing the essential building blocks for myofibrillogenesis. It is very unlikely that MIR and its binding proteins directly promote tropomyosin translation, since it takes 3-4 days after MIR transfection before increased tropomyosin expression and myofibril formation is observed in the mutant heart cells.

Recent studies show that short double-stranded RNA duplexes trigger post-transcriptional gene silencing and can also induce epigenetic silencing of genes at the level of transcription [52]. They can also interact at promoter regions and can activate or repress gene expression [53] or affect translation without affecting transcription [54].

While studies on siRNAs and miRNAs were extensively conducted in a wide variety of systems, comparatively, no remarkable studies were reported on small RNAs especially those that are similar to MIR. Recently, Makarev et al. [55] identified several miRNAs and small RNAs from adult newt eye, 42 of which have no similarity with known miRNAs or piRNAs. Our studies also clearly suggest that our MIR has no sequence homology with any of these known RNAs. Furthermore, none of the known small RNAs, to our knowledge, have been shown to regulate myofibrillogenesis similar to MIR. However, miRNAs on the other hand have been implicated as regulators of transcription as well as translation in a wide variety of systems [54,56].

Although such reports indicate the regulatory role of different small RNAs at the transcription and/or translational levels, the underlying molecular mechanisms, however, are still poorly understood. Whatever the final mechanism of action of MIR turns out to be, it is very clear that MIR promotes tropomyosin synthesis and myofibrillogenesis in the mutant hearts and is essential for tropomyosin expression and myofibrillogenesis in normal hearts. We are conducting several experiments to isolate functional mouse/human homologues of MIR for further characterization. We assume also that MIR, or an MIR-type functional homologue, is involved in cardiomyocyte precursor cell differentiation in all vertebrate species.

## Competing interests

The authors declare that they have no competing interests.

## Authors' contributions

CZ designed and carried out many of the experiments in the study. He cloned and sequenced the full length DNA for the MIR and conducted the real-time RT-PCR experiments. PJ worked on the molecular biology and tissue culture experiments contributing significantly to both areas. XH was involved in the planning of the experiments and the immunofluorescent studies for tropomyosin in the MIR rescued hearts. He also played a major role in writing the manuscript for publication. GFS participated in the analysis of the different tropomyosin isoforms in the determination of myofibril protein expression in the embryonic hearts. GA conducted and oversaw MIR rescue experiments and performed many of the immunofluorescent studies as well as real-time PCR studies. MPA provided guidance in the selection and data presentation and analysis. He also wrote significant portions of the manuscript. JW participated in the analysis of many of the immunofluroescent images and participated in writing the immunofluorescent sections of the manuscript. SL designed the mating protocols for the animal colony to obtain the time-staged embryos/tissues for the studies. In addition, she oversaw the preparations of the figures as well as the text of the manuscript. DKD was involved in the original design of the experiments for the study and participated in organizing the data to be included in the manuscript. In addition, he contributed significantly to the writing of the manuscript for publication. LFL served as the Principle Investigator on the study and the grants that supported the study. He oversaw all of the experiments in the study and coordinated the research activities of the study. He wrote/edited the entire manuscript and is the communicating author of the paper. All authors read and approved the final manuscript.
